# Development and pilot of a drug use and HIV stigma reduction training for Tajik migrants who work in Moscow: The SRI-AVLOD intervention

**DOI:** 10.21203/rs.3.rs-9358383/v1

**Published:** 2026-04-23

**Authors:** Mary Ellen Mackesy-Amiti, Judith A. Levy, Leslie D. Williams, Jonbek Jonbekov

**Affiliations:** University of Illinois Chicago; University of Illinois Chicago; University of Illinois Chicago; PRISMA Research Center

**Keywords:** intersectional stigma, intervention development, drug use, HIV, Tajik, labor migrant

## Abstract

**Objective:**

Tajik labor migrants who inject drugs while working in Moscow are subject to the negative effects of stigma within the diaspora community in Russia. They are also at risk of acquiring HIV, which further compounds their stigmatization. An intersectional intervention to reduce both drug-related and HIV-related stigma within the Tajik diaspora community is needed to prevent adverse social and health effects among its members who inject drugs.

**Methods:**

We conducted a series of focus groups and interviews with Tajik migrant workers and health workers who treat migrants in Moscow to inform the development of a five-session anti-discrimination training for Tajik community leaders. The training sessions are designed to educate leaders about substance use disorder and HIV and prepare them to act as change agents in reducing stigma toward both disorders within the Moscow Tajik diaspora community. We pilot tested the Stigma Reduction Intervention Approach Via Leaders of Diaspora (SRI-AVLOD) for cultural acceptability and feasibility with two groups of traditional migrant community (*avlod*) leaders while residing in Tajikistan before returning to Moscow for work. Measures of drug use and HIV-related stigma were administered prior to the first and after the final training session. We collected participant feedback on the acceptability and feasibility of the intervention and conducted a brief follow-up interview post-training to assess participants’ experience in delivering the anti-stigma messages to their community.

**Results:**

Participant ratings indicated high acceptability and satisfaction with the training. Measures of stigmatizing attitudes among the *avlod* leaders were significantly reduced following the intervention. In follow-up interviews, participants reported that they delivered the anti-stigma message to an average of 16 people.

**Conclusion:**

The SRI-AVLOD intervention proved feasible and acceptable to Tajik community leaders and was judged to have considerable potential for effective diffusion within the migrant diaspora community.

## Introduction

Stigma toward international migrants is a global phenomenon with significant consequences for individuals and public health (1, 2). Tajikistan, a small country in Central Asia with a high unemployment rate and an ongoing drug epidemic,(3–5) exports more than a million temporary labor migrants to Russia for work each year.(6, 7) In 2024, international remittances from Tajik migrants to their home country, mostly from Russia, accounted for 49% of the country’s GDP and supported 21% of households.(8) Depending in part on gender, most Tajik migrants find employment in the Russian Federation working in construction, trade, agriculture, maintenance, and the domestic sector (9, 10). As true for labor migrants in many countries, life in Russia for Tajik workers is often hard. In addition to taking on Russia’s most dangerous and undesirable jobs (11), they often are the focus of Russian hostility and violence,(12) endure poor living conditions, and have little or no access to Russian health care if sick or injured.(13) At the same time, some unknown number of Tajik migrants having entered Russia illegally must constantly hide from Russian immigration authorities.

These factors, along with the psychosocial stress and separation from family and friends, can coalesce to produce emotional distress including depression and feelings of loneliness that can lead to initial or accelerated drug use, encourage risky sexual and/or drug behavior, discourage HIV testing, and result in poor HIV medication adherence and treatment outcomes (14–18). A recent study provides evidence that Tajik migrants with HIV working in Russia are at increased risk of late presentation to treatment.(19) In addition, most Tajik migrants in Moscow reside in close-knit diaspora communities where those who inject drugs, especially if they acquire HIV, are subject to extensive censure and marginalization from their non-using Tajik peers.(20, 21) Such social exclusion and adverse treatment while in diaspora can compound or exacerbate the emotional distress commonly associated with being a migrant.

No interventions currently exist to mitigate the social beliefs and behavior within a diaspora migrant community that underpin and reinforce stigma related to drug use and HIV. To address this unmet need, we investigated the character of stigma in the Tajik labor migrant community and the effects of intersecting forms of stigma on the health and well-being of Tajik migrant people who inject drugs (PWID) and people living with HIV (PLWH) in diaspora. Rather than focusing on the largely intractable attitudes of a host society, we direct our intervention efforts to changing stigmatizing attitudes and behavior within the Tajik diaspora community itself. Using a community participatory approach to inform the intervention, the model that we developed draws upon Tajikistan’s traditional avlod leadership arrangement that forms the basic unit of Tajik social structure. The avlod system of authority in Tajikistan is based on Tajik tradition that accords a cadre of highly respected community leaders command and influence over the behavior and beliefs of their extended kin and village members.(22, 23) We consulted avlod leaders of the Moscow Tajik diaspora community as key participants in developing a network-based host-country leadership HIV and drug-related stigma intervention. Drawing on the insight and findings gained through formative research, we developed and pilot tested the acceptability and feasibility of the Stigma Reduction Intervention Approach Via Leaders of Diaspora (SRI-AVLOD) model. This model is specifically designed to counter the negative effects of both HIV-related and drug-related stigma that can propel risk behavior and impede prevention and treatment among Tajik labor migrants who inject drugs while working in a host country.

Close-knit Tajik communities in diaspora are ideal socio-cultural settings for implementing a successful HIV and drug use stigma-reduction intervention. As our prior research shows: (1) Tajik diaspora communities have clear and approachable leaders who are highly respected and can shift perceived norms and thereby influence others to adopt positive behavioral change; and (2) members of diaspora communities tend to interact closely with each other and with this leadership. Consequently, new norms and behaviors can be effectively diffused and promoted within and across migrant social networks if endorsed by leaders whom the members of these networks trust. SRI-AVLOD builds on both advantages by opening up conversation by *avlod* leaders within the Tajik diaspora community about the direct effects of stigma on its members who inject drugs and/or are living with HIV and its indirect effects on their family, friends, and the community at large. SRI-AVLOD recruits and trains Tajik diaspora community leaders as potential agents of change in reducing drug-related and HIV-related stigmatizing beliefs and actions within the Tajik community that negatively affect risk behavior, prevention, and treatment among its members who inject drugs.

Our research efforts build on the integration of 4 conceptual models: 1) community participatory research in which stakeholders and those affected by an intervention are included as partners in contributing expertise and sharing in the decision-making concerning the model’s development and delivery; 2) Social cognitive theory that considers the influence of observational learning, modeling, and perceived self-efficacy when developing effective interventions to promote sustained and translatable behavior change (24); 3) Social Identity Theory, which posits that people are stigmatized when considered to be members of an “out-group” with few similarities to one’s “in-groups” (24), and conversely that increasing the salience of commonalities with one’s “out-group” members can help to reduce stigma (25); and 4) Diffusion of Innovation Theory that posits that new information, norms, and/or attitudes can be disseminated and integrated into a setting or group via a small number of influential individuals.(26)

## The SRI-AVLOD Intervention Leadership Model

Upon migrating to Russia and other destinations, labor migrants join other Tajiks who have migrated before them to create close social ties, provide mutual support, and establish a migrant network of *avlod* leaders abroad. These powerful leaders and the networks of migrant workers whom they influence offer a strong, well-established social structure through which to effect normative and behavioral change.

The SRI-AVLOD intervention begins by targeting possible stigmatizing beliefs, attitudes, and behavior held by *avlod* leaders toward the diaspora community’s PWID and PLWH. The goal is to positively change any negative beliefs and behavior that these influential leaders might hold toward people living with these two disorders with the expectation that, in turn, they will use their social network leadership to similarly educate other Tajiks. Comprised of four educational sessions, the SRI-AVLOD intervention is delivered to groups of 6–8 *avlod* leaders. It includes an overview of the challenges that PWID confront in ending or practicing safer drug use and how stigmatizing attitudes, norms, and behavior can hinder this process and lead to heightened HIV risk behavior, undermine HIV prevention, and discourage HIV testing and/or seeking treatment if needed. At the end of the four sessions, each leader is asked as homework to select a venue or other opportunity to speak with other Tajik migrants in discussing and promoting positive normative and behavioral change within the Tajik diaspora community toward its members who are PWID and/or PLWH. Evaluation measures in this preliminary pilot assess behavioral and attitudinal change post intervention among the participants, success in discussing new norms and beliefs with other Tajik migrants, and participant satisfaction with and perceived usefulness of the training including its feasibility in bringing about positive community change.

## Methods

### Phase 1 Formative Research: Development of the SRI-AVLOD curricula and manual

We explored the nature and effects of stigma specifically related to drug use and HIV

Separate focus group discussion guides were developed and tailored to fit each of the three categories of FG participants. In addition to discussing HIV and drug-related topics relevant to each group’s organizational, workplace and/or community experience, attention was directed to key topics related to stigma and stigmatizing attitudes and behavior that participants may have observed or personally enacted toward people with HIV and/or drug use disorder.

Both the focus group discussions and in-depth interviews explored possible Tajik community and organizational-level facilitators available to support the SRI-AVLOD HIV and drug use stigma reduction intervention, possible impediments to its implementation, and cultural mores and practices to consider in developing and mounting it. Importantly, since HIV-related and drug use-related stigma enacted towards women is tied to traditional gender expectations in Tajikistan and typically is more severe toward them than toward men (27, 28), we explored potential gender differences that could affect the messaging and delivery of the intervention.

From the findings, the investigators collaboratively developed the SRI-AVLOD training and intervention manual. The 4-session training was designed to reduce both HIV and substance-use related stigma among *avlod* leaders and later among Tajik community members via social network diffusion. Curricula included the biological and trauma-related etiology of substance use disorder, dispelling false beliefs about HIV transmission, increasing understanding and empathy for PWID and PLWH, and training *avlod* leaders in effective communication strategies. The stigma experiences that PWID reported informed the creation of relatable vignettes to explain the serious consequences of drug-related and HIV-related stigma. To address common misconceptions about both HIV and drug use, we developed a “Fallacies and Facts” format within the curricula to debunk false beliefs and replace them with corresponding scientific fact. The draft manual was presented to the study’s Tajik Community Advisory Board, from which feedback was requested and incorporated as needed.

### Phase 2: Pretest of the SRI-AVLOD Intervention

We delivered the 4 training sessions to 2 groups of 5–7 diaspora community leaders in Tajikistan, one held in the capital city of Dushanbe and one in GBAO. We presented each session in its entirety, followed by participant discussion and critique. A focus group guide helped to direct the discussion to key topics such as attendees’ comfort with the content of the sessions, their cultural appropriateness for use with Tajik migrants, the perceived usefulness of the sessions’ content in reducing HIV-related and drug use-related stigma, how the information could best be disseminated through their networks, and suggested modifications. The sessions were audio-taped, transcribed, and translated for analysis. The results were used to further develop the SRI-AVLOD intervention and its leadership training sessions. The revised manual was presented to the Community Advisory Board for requested feedback. An outline of the finalized manual is available in Appendix A (see Additional File 1).

### Phase 3: Pilot testing the Intervention

The intervention was delivered to two groups of eight diaspora community leaders. At the start of the first session, we collected sociodemographic information from the participants, including gender, age, occupation, and other social roles held in the community, as well as measures of leaders’ knowledge about HIV, and attitudes regarding Tajik PWID and PLWH. At the end of the program, we readministered the measures of HIV knowledge and attitudes regarding PWID and PLWH and collected program evaluations. We also asked participants to describe their plans for speaking with Tajik migrants with whom they interact in a leadership role to discuss and promote positive normative and behavioral change within the Tajik diaspora community toward its members who use drugs or are living with HIV. We conducted a brief telephone interview with participants 4–6 weeks after the last session to ask them about their interactions with community members (e.g. how many people they talked to about HIV and/or drug use, whether individually or in groups), how receptive community members were to their message, and their opinions about how well the intervention prepared them to have these discussions.

### Measures

Both process and training outcome measures were adapted from existing English-language measures. Items were translated into Tajik by a native speaker and independently back-translated to English for verification. To eliminate unreliable or confusing items, we pretested the draft instruments with five native Tajik speakers using cognitive interviewing to solicit feedback. After making revisions, a survey was administered to 60 returned migrants to test performance of the instruments including internal reliability and cross-correlations. Items with poor reliability were removed or replaced if a wording change was deemed appropriate. The final measures are available in Appendix B (see Additional File 2).

Familiarity with Drug Use Disorder and HIV. These two measures, based on the Level of Contact Report (29, 30), each consist of a series of twelve yes/no items regarding familiarity with HIV or drug use disorder (DUD). The items are summed to form an index ranging from 1 (“I have never [observed | known] a person with a [drug use disorder | HIV]”) to 12 (“I have a [drug use disorder | HIV]”).

HIV knowledge. The HIV knowledge measure included 8 items that described behaviors that respondents were asked to identify as safe or unsafe with regard to transmission of HIV and 8 true/false items pertaining to HIV infection, testing, and treatment. Items were scored as correct or incorrect with responses of “unsure” scored as incorrect. The total score is calculated as the number of items correct (*alpha* = 0.80).

Brief Opioid Stigma Scale (BOSS). (31) This scale includes four statements of things that “most people believe” about a person who is addicted to opioids (measuring perceived societal stigma), and four corresponding statements of things that “I believe” (measuring individual stigmatizing attitudes), e.g., “[I / Most people] believe that a person who is addicted to opioids cannot be trusted.” We added two additional culturally relevant statements, “[I / Most people] believe that a woman who is addicted to opioids is promiscuous.”] Items are rated on a scale from 1 “strongly disagree” to 5 “strongly agree” (3 = unsure). Item ratings on each subscale are summed to produce a score ranging from 5 to 25 (*alpha* = 0.64 for individual stigmatizing attitudes; 0.62 for perceived societal stigma).

Stigmatizing Attitudes towards PLWH Scale - SAT-PLWH-S. (32) The adapted scale consists of 20 positive and negative statements (reduced from 27) to assess stigmatizing attitudes toward people living with HIV rated on a Likert scale from 1 “strongly disagree” to 5 “strongly agree.” We changed “AIDS” to “HIV” and we made some other minor changes to the wording of some items. Negative statements are reverse scored so that higher scores indicate a more positive attitude. Item ratings are averaged to produce a continuous score ranging from 1 to 5. After eliminating 7 items with poor reliability, *alpha* = 0.90.

Attribution Questionnaire. This measure was adapted from Corrigan et al. Attribution Questionnaire Short Form (AQ-27) (33). After reading a vignette describing a person with opioid use disorder (OUD), participants respond to a series of 20 statements representing constructs of personal responsibility beliefs, pity, anger, fear, helping and avoidance, and support for coercion and segregation. Each item was rated on nine-point semantic differential type scale (1 = “not at all”, 9 = “very much”). The scale score was computed as the average of item ratings with positive items reversed so that higher scores represent more negative attitudes (*alpha* = 0.89).

Social Distance. This measure was adapted from Link et al. (34) to measure desire for social distance. After reading a vignette about a person in recovery for opioid use disorder, participants rated their willingness to engage in 7 behaviors (e.g., sharing an apartment, recommending for a job). Items were rated on a 4-point Likert scale (1 = “definitely willing”, 4 = “definitely unwilling”). Next, participants were asked to respond to the same items after learning that the person also is receiving HIV medication. Subscale scores were averaged to form a social distance score. Internal reliability was excellent for both the initial ratings (*alpha* = 0.86) and the subsequent ratings (*alpha* = 0.93).

## Results

### Pilot Participants.

Characteristics of *avlod* leader participants in piloting the intervention are shown in Table 2. Ages ranged from 36 to 55 (mean = 48). Most participants (82%) were well educated with some level of higher education or having completed a college degree. The number of lifetime trips to Russia ranged from 2 to 8 (mean = 4.6), and the cumulative number years living in Russia ranged from 4 to 11 (mean = 7.4). All participants worked and resided in Moscow with some form of legal status. Slightly less than half knew someone with HIV.

*Process variables* measured participants’ assessment of the SRI-AVLOD training and curricula that they received in terms of its role in increasing their own knowledge and positive values and later in terms of their perceptions of the degree to which it provided valuable messaging in informing their role as stigma-reduction change agents. Participants rated the training highly on all 5 assessment variables (Table 3). No differences were found between Tajikistan locations or by sex.

### Change in Avlod Leader Stigma Opinions and Beliefs Post Leadership Training (Table 4).

HIV knowledge scores increased significantly following the intervention, and measures of stigmatizing attitudes related to drug use and HIV significantly decreased. There were no significant differences between geographic locations or by sex. Perceived societal stigma scores did not change significantly.

### Follow-up Qualitative Interviews.

Participants reported delivering anti-stigma messaging to an average of 16 people including both individuals and groups. All reported feeling very confident talking to other Tajiks about stigma related to substance use and HIV in part because of their position as a trusted leader in the community, but they also credited the training.

“The training helped me consolidate the material on discrimination, the importance of personal communication in breaking down prejudices, and the understanding that every conversation is a step toward a more just and humanized society.”

– Dushanbe [15]

Comments by *avlod* leaders indicate that generally their messages were received positively. Some conversations shifted from initial surprise or caution about discussing such stigmatized disorders to openness. Tajiks with whom they spoke commonly reported learning about stigma and its harmful effects to be eye-opening, and they acknowledged the presence of discrimination within Tajik society including in diaspora. People initially agreed or came to agree through discussion that drug use disorder (DUD) and HIV should be treated as health conditions and that they understood the need for empathy and community support. Overall, from the perspective of the *avlod* leaders, the anti-discrimination discussions inspired positive attitude changes. A sample of *avlod* leader comments are presented below.

## How did people react to what you told them?

“People accept that we need to change our attitude toward drug users and people with HIV because these people could be useful members of our community.”

– GBAO [4]

“People of all ages responded very humanely. I was a little nervous in the beginning, but when I started speaking, I saw many listening attentively and nodding in agreement. Some were surprised having never considered that stigma isn’t just a word but a real pain for others.”

– Dushanbe [9]

“Most people were willing to listen to my message and responded receptively. Many-over 90%-said they were glad to receive this information. They said the topic was useful and very timely. During the conversation, I sensed that people genuinely lack reliable knowledge about stigma and its consequences. When information is presented calmly, with real-life examples, people become more open and interested.”

– Dushanbe [14].

Several participants indicated that people were initially skeptical.

“Surprised that I am telling them to be tolerant and empathetic toward people with drug use disorder and HIV. But explaining to people that drug use disorder and HIV are just diseases that need to be treated and with the support of community, relatives and friends, people could live a better life with drug use disorder and HIV. Everybody came to agree that the situation with stigma needs to be changed. People with drug use disorder and HIV deserve to be part of our community and to be happy.”

– GBAO [6]

“At first, the conversation was a bit tense-some relatives thought addiction was simply a weakness. I explained that it was a disease that required support, not judgment.”

– Dushanbe [9]

“A few were initially cautious, but after explanations and real-life examples, they became more interested.”

– Dushanbe [12]

“What I remember most was that at the beginning of the conversation, most of my discussion partners advocated for harsher and more restrictive measures against people who use psychoactive substances and those living with HIV. However, gradually, they began to understand more deeply that discrimination does exist in our community, and it is important to find ways to prevent it.”

– Dushanbe [14]

## Discussion

Our formative research with Tajik labor migrants, diaspora community leaders, healthcare workers, and PWID was instrumental in shaping a culturally relevant intervention to train *avlod* leaders on reducing intersectional stigma related to drug use and HIV in their social networks and the diaspora community. Through interviews and focus groups, we identified and then presented during the *avlod* leader training concrete examples of stigmatization, discrimination, and the consequences of stigma as well as countering damaging misconceptions about HIV and people who use drugs.

The pilot test of the intervention demonstrated the SRI-AVLOD model’s feasibility and acceptability among Tajik *avlod* leaders who work in Moscow. HIV knowledge significantly increased among these leaders following the training while stigmatizing beliefs and attitudes regarding people living with HIV or DUD significantly decreased. In the following weeks, participants actively engaged in conversations with people in their communities individually and in groups about HIV, drug use, and related stigma. They reported that the network members with whom they discussed HIV-related and drug-related stigma indicated that they found the information acceptable, appropriate, and useful, as they themselves did at the end of their training. They reported positive engagement experiences with their networks that they perceived helped Tajik diaspora community members to recognize the harm caused by stigma and to shift their attitudes to be more empathetic and inclusive.

## Limitations

We originally conceived conducting the stigma reduction trainings in Moscow, however international events transpired that necessitated modifying the protocol to conduct the trainings with returning migrants in Tajikistan. This resulted in variability in the lag between the training and applying what was learned in the diaspora environment. Some of the participants had not yet returned to Moscow when the follow-up interviews were conducted, and instead practiced what they had learned with community members in their home country.

Internal consistency reliability of the BOSS scales measuring individual stigmatizing attitudes and perceived societal stigma against opioid use did not reach an acceptable level and would not be recommended for use in future research with this population. However, the Attribution Questionnaire for OUD and the Social Distance Scale performed well.

This research is a pilot study that was conducted among a small sample, and without a comparison group. Future research should build upon the preliminary findings presented here and address these limitations by conducting a randomized trial of the SRI-AVLOD intervention to test its efficacy experimentally.

## Conclusion

Overall, the SRI-AVLOD Intervention Leadership Model holds considerable promise for reducing HIV and drug-related stigma among members of the Tajik Moscow diaspora community and also likely at home in Tajikistan.

## Supplementary Files

This is a list of supplementary files associated with this preprint. Click to download.

- AdditionalFile1.pdf
- AdditionalFile2.pdf

Additional Material

Additional File 1.pdf: Appendix A. Outline of the finalized manual for SRI-AVLOD

Additional File 2.pdf: Appendix B. Measures used in SRI-AVLOD pilot

## Figures and Tables

**Table 1:** Formative research participant categories (N = 83)

Participants	Description	Number of participants
Migrant workers	5 focus groups representing 5 regions	25
Diaspora leaders	5 focus groups representing 5 regions	25
Health workers	3 focus groups representing 3 clinics	15
PWID	In-depth interviews, 3 per region	15
Health clinic directors	In-depth interviews, 1 per clinic	3

**Table 2:** Sociodemographic characteristics of pilot study participants (N = 16)

Variable	n	%
Sex		
Male	12	75%
Female	4	25%
Marital status		
Married	11	69%
Divorced	5	31%
Education		
Secondary	3	19%
Some higher education	3	19%
Completed university	10	63%
Employment		
Construction	6	38%
Bazaar	5	31%
Service organization	5	31%
Diaspora organization committee member		
No	10	63%
Yes	6	38%
Legal status in Russia		
Citizen	1	6%
Applied for citizenship	5	31%
Residency permit	3	19%
Temporary work permit	7	44%
Know someone with HIV		
No	9	56%
Yes	7	44%

**Table 3:** Post-intervention process measures

Item/Scale	Mean rating
How satisfied are you with the training you received and the practices covered?	4.8
How convincing were the presenters?	4.8
Was the training well organized and implemented?	4.6
How satisfied are you with the content of the training and the practices covered?	4.7
How satisfied are you with the level of complexity of the training and the practices covered?	4.5
How comfortable are you with the practices contained within the training?	4.7
**TOTAL Acceptability**	**4.7**
How useful are the information and practices from the training to you for community leadership?	4.7
To what extent do you expect to be able to incorporate the concepts and techniques from the training into your leadership activities?	4.3
**TOTAL Feasibility**	**4.5**
How relevant are the information and practices to your population?	4.4
How well do the information and practices from the training fit with your overall approach to community leadership?	4.2
**TOTAL Appropriateness**	**4.3**
I learned something new in this training.	4.9
The information presented was easy to understand.	4.8
I feel confident that I can raise the issue of stigma and how it affects people who use drugs with people in my community.	4.7
I feel confident that I can discuss the issue of stigma with people in my community.	4.8
I have a plan for meeting with people in my community to discuss the issue of stigma.	4.9
Reducing stigma against people who use drugs is a worthwhile objective.	4.8
I would recommend the SRI-AVLOD training to people I know.	4.8
**TOTAL Usefulness**	**4.8**

Note: Items were rated on a 5-point scale from 1 = not at all to 5 = extremely

**Table 4:** Pre- and post-intervention HIV knowledge and stigma measures

Measure	Pre-intervention		Post-intervention			
	mean	SD	mean	SD	z^a^	p
HIV knowledge	8.5	3.6	15.9	0.34	8.47	< 0.001
HIV stigmatizing attitudes	55.4	10.9	38.1	4.2	−6.19	< 0.001
DUD stigmatizing attitudes	15.6	3.7	10.8	1.7	−5.68	< 0.001
Societal DUD stigma	15.9	4.2	14.1	3.7	−1.90	0.057
Social distance DUD	19.8	3.3	13.7	1.7	−8.14	< 0.001
Social distance DUD + HIV	18.6	3.7	11.3	2.4	−9.39	< 0.001
Attribution Questionnaire	4.8	0.9	2.3	0.3	−10.71	< 0.001

a coefficient significance test from mixed effects model including sex, age, and location as covariates

## Data Availability

Data analyzed during the current study are available on Open Science Framework (OSF) [Project overview: [https://osf.io/2nbpd/overview](https:/osf.io/2nbpd/overview); Pilot data: [https://doi.org/10.17605/OSF.IO/A7KNW](https:/doi.org/10.17605/OSF.IO/A7KNW)].

## Abbreviations

AQ-27: Attribution Questionnaire Short Form
BOSS: Brief Opioid Stigma Scale
DUD: drug use disorder
GBAO: Gorno-Badakshan Autonomous Oblast
HIV: human immunodeficiency virus
OUD: opioid use disorder
PLWH: people living with HIV
PWID: people who inject drugs
RRP: Region of Republican Subordination
SAT-PLWH-S: Stigmatizing Attitudes towards PLWH Scale
SRI-AVLOD: Stigma Reduction Intervention Approach Via Leaders of Diaspora
